# Enhancing genome assemblies by integrating non-sequence based data

**DOI:** 10.1186/1753-6561-5-S2-S7

**Published:** 2011-05-28

**Authors:** Thomas N Heider, James Lindsay, Chenwei Wang, Rachel J O’Neill, Andrew J Pask

**Affiliations:** 1 Department of Molecular and Cellular Biology, University of Connecticut, 06269, Storrs CT, USA; 2 Department of Computer Science and Engineering, University of Connecticut, 06269, Storrs CT, USA; 3 Faculty of Veterinary Science, The University of Sydney, NSW 2006, Australia; 4 Current address: ARC Centre of Excellence in Bioinformatics, Institute for Molecular Bioscience (Building #80), The University of Queensland, Brisbane, QLD 4072, Australia

## Abstract

**Introduction:**

Many genome projects were underway before the advent of high-throughput sequencing and have thus been supported by a wealth of genome information from other technologies. Such information frequently takes the form of linkage and physical maps, both of which can provide a substantial amount of data useful in *de novo* sequencing projects. Furthermore, the recent abundance of genome resources enables the use of conserved synteny maps identified in related species to further enhance genome assemblies.

**Methods:**

The tammar wallaby (*Macropus eugenii*) is a model marsupial mammal with a low coverage genome. However, we have access to extensive comparative maps containing over 14,000 markers constructed through the physical mapping of conserved loci, chromosome painting and comprehensive linkage maps. Using a custom Bioperl pipeline, information from the maps was aligned to assembled tammar wallaby contigs using BLAT. This data was used to construct pseudo paired-end libraries with intervals ranging from 5-10 MB. We then used Bambus (a program designed to scaffold eukaryotic genomes by ordering and orienting contigs through the use of paired-end data) to scaffold our libraries. To determine how map data compares to sequence based approaches to enhance assemblies, we repeated the experiment using a 0.5× coverage of unique reads from 4 KB and 8 KB Illumina paired-end libraries. Finally, we combined both the sequence and non-sequence-based data to determine how a combined approach could further enhance the quality of the low coverage *de novo* reconstruction of the tammar wallaby genome.

**Results:**

Using the map data alone, we were able order 2.2% of the initial contigs into scaffolds, and increase the N50 scaffold size to 39 KB (36 KB in the original assembly). Using only the 0.5× paired-end sequence based data, 53% of the initial contigs were assigned to scaffolds. Combining both data sets resulted in a further 2% increase in the number of initial contigs integrated into a scaffold (55% total) but a 35% increase in N50 scaffold size over the use of sequence-based data alone.

**Conclusions:**

We provide a relatively simple pipeline utilizing existing bioinformatics tools to integrate map data into a genome assembly which is available at http://www.mcb.uconn.edu/fac.php?name=paska. While the map data only contributed minimally to assigning the initial contigs to scaffolds in the new assembly, it greatly increased the N50 size. This process added structure to our low coverage assembly, greatly increasing its utility in further analyses.

## Background

**Figure 1:**
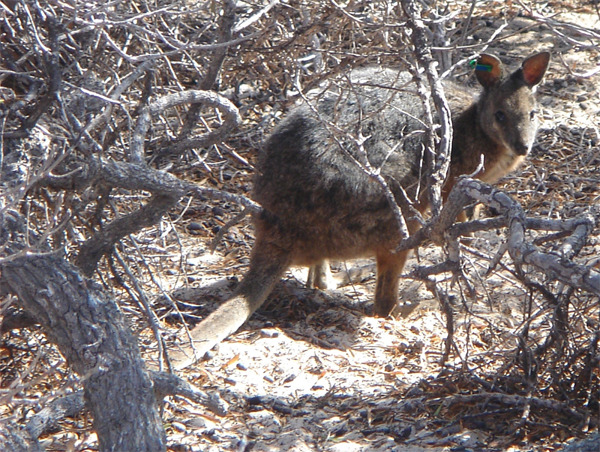
**The tammar Wallaby (*Macropus eugenii*).** An adult female tammar wallaby of Abrolhos Island origin, Western Australia. Females weigh 4-6kg and males 5-9kg.

**Figure 2:**
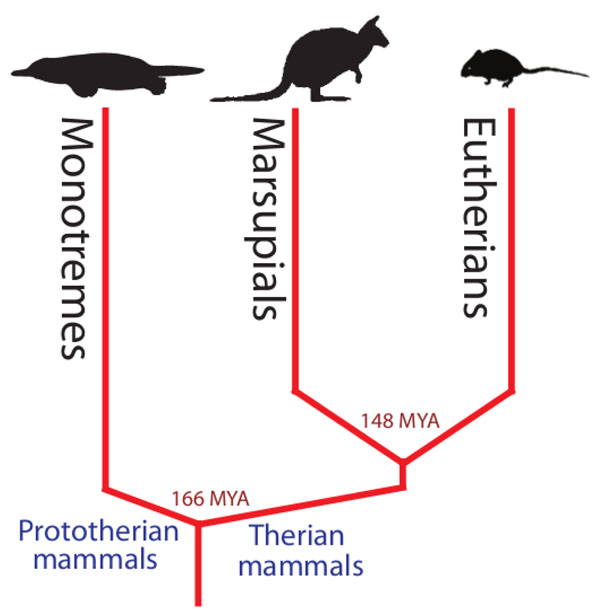
**Phylogenetic tree of the three extant mammalian lineages.** Marsupials form a separate lineage from the eutherian mammals and last shared a common ancestor approximately 148 million years ago making them powerful species for comparative genomics.

The tammar wallaby (*Macropus eugenii*) belongs to the marsupial lineage within the Class Mammalia (Figure 1). Marsupials differ from eutherian mammals in that they give birth to relatively altricial young that complete much of their development external to the mother, attached to a nipple often enclosed in a pouch [1]. These developmental novelties make marsupials ideal models for examining and manipulating early stages of mammalian development and reproduction [2-4] otherwise not possible in model eutherian mammal species (such as mouse and rat) where development occurs largely *in utero*. In addition to their developmental differences, marsupial mammals are unique in that they have been evolving independently of eutherian mammals for over 148 million years [5] (Figure 2). From a genomics perspective, this makes them ideal for comparisons with eutherian mammals to isolate important functional regions of the genome; 148 million years of divergent evolution is sufficient for non-functional DNA to no longer retain homology, while functional DNA can be easily identified between the lineages [6-8]. For example, cross comparisons between marsupial and eutherian genomes have enabled the identification of important coding as well as non-coding (including elusive promoter) elements [8-10].

The arrangement of the tammar wallaby genome is unique, with the entire 2.7 Gb genome organized into seven pairs of autosomes and an X and Y [11,12]. However, the X is relatively small in the tammar and the Y is tiny. It has been proposed that the tammar X represents the ancestral therian X chromosome, which has undergone several additions in the eutherian lineage. Likewise the Y in marsupials is thought to represent a minimal mammalian Y [13,14]. The centromeres of the tammar chromosomes are also quite different from that of their eutherian relatives. Similar to centromeres in rice [15], the centromeres of the tammar are small, encompassing ~420kb, and are comprised of a heterogeneous repeat structure of interspersed satellites and centromeric retroelements [16]. In addition, the alternative reproductive strategy of the tammar has placed different evolutionary pressures on the genome. Most notably, genomic imprinting, an epigenetic phenomenon in eutherian mammals thought, in part, to regulate fetal growth and nutrition *in utero* affects fewer genes and is less complex in marsupials [17,18]. All of these features combined, make the tammar wallaby genome a particular interesting resource from an evolutionary as well as a developmental point of view.

A white paper to sequence the genome of the tammar wallaby was funded in 2004, in a joint venture between the National Human Genome Research Institute (National Institutes of Health) and the Australian Genome Research Facility Ltd. (http://www.genome.gov/Pages/Research/Sequencing/SeqProposals/WallabySEQ.pdf) to produce a 2× coverage genome. By the time the project was initiated, large amounts of non-sequence based data had already been collected to supplement Sanger sequence reads. Work continued with the non-sequence based data as a way to enhance the genome assembly. Subsequently, a comprehensive map of the wallaby genome was developed that integrated physical, linkage and synteny maps with 14,429 markers spanning the tammar genome [19]. This abundance of markers was greatly enhanced by synteny maps constructed between the tammar wallaby and the genome of the opossum (another marsupial species). While a large number of model species with low coverage genomes already have genomic maps, including *Canis familiaris, Felis catus* and *Ovis aries*, none have such an extensive map as the wallaby, with the next largest map found in the dog project, including 4,249 markers [20]. A well-developed map plays an important role in creating an accurate representation of the genome by providing a structure to which many smaller contigs can be attached and ordered to produce a whole chromosome. Thus, an extensive genomic map can help overcome many of the limitations of a low coverage genome and provide a platform for researchers to investigate repetitive regions, gain insight into chromosomal evolution and help to develop regulatory pathways that may depend on proximity for activity not possible without an enhanced assembly.

## Methods

### Integrating the nonsequence-based data

The tammar virtual genome maps were previously constructed for each tammar wallaby chromosome, combining physical, linkage and synteny data [19]. Maps consisted of markers identified by their opossum Ensembl IDs, their gene names and physical location on the tammar wallaby chromosomes. The Ensembl IDs were then used to retrieve the opossum gene sequence (exons and introns) from Ensembl using the published interface provided from its website. Sequences were then aligned against the assembled contigs from the tammar wallaby 1.2 assembly using BLAT [21] to identify the location of each marker with the highest scoring match being used in the final scaffold. As an initial scaffolding of the contigs in the genome had been previously accomplished using SOLiD mate pair reads, the scaffolds generated from that analysis were treated as the initial contigs used in the following analyses. The output from BLAT was used to create a contig file for Bambus, using the file specifications outlined at http://sourceforge.net/apps/mediawiki/amos/index.php?title=Bambus_Manual. To generate the mate-pair file for Bambus [22,23], all possible marker combinations were identified for a set distance using the physical map locations on tammar wallaby chromosomes. This process started with a 5MB interval and was repeated in 1 MB increments to a 10 MB distance between the two markers, generating a total of 6 pseudo mate-paired libraries. The statistics of how this improved the assembly (Table 1) were all obtained from the Bambus .stats output file.

**Table 1:** Summary statistics for scaffolding of sequence and nonsequence based data

Run Included	Number of paired-end reads	Total scaffold span	N50 scaffold span	Number of scaffolds	Percentage of original contigs included into scaffolds
**Initial Assembly**	---	---	36 KB	277,711	0.0 %
**Virtual map**	173,294	3204 MB	39 KB	271,687	2.2 %
**4kb library**	8,415,542	3069 MB	49 KB	165,909	40.2 %
**8kb library**	11,718,457	3177 MB	52 KB	202,026	27.2 %
**Illumina libraries**	20,133,999	2829 MB	78 KB	129,290	53.4 %
**All data**	20,407,293	2534 MB	105 KB	124,099	55.2 %
**Ideal Genome**	---	2700 MB	---	8	---

This table shows the relative contributions that each library makes to reducing the number of scaffolds and increasing the N50. All data are derived from the Bambus output statistics file. The left column indicates the data set used to enhance the assembly with Bambus. Number of pared end reads indicates the total number of paired data points used for each data set to enhance the assembly. The total scaffold span provides an indirect assessment of the genome size based on the assembly and was used by Bambus to calculate the N50. The N50 indicates the size of the smallest contig in the smallest set of contigs that add up to 50% of the size of their respective total scaffold span. The number of scaffolds indicates the number of independently ordered regions in our assembly. The reduction in this number with the integration of each library indicates the integration and ordering of the original contigs into larger scaffolds. The total number of scaffolds generated from the assembly is listed and the percentage reduction is from the initial number of contigs present in the input library. The bottom row lists the ideal genome size (2.7 GB) and number of contigs (one for each chromosome = 8).

### Integrating the sequence-based data

In addition to the physical map, there was also a wealth of information from next generation sequencing platforms that we wanted to integrate into the tammar genome assembly. The Illumina paired-end reads were mapped against the tammar wallaby genome using the short read mapping program Bowtie [24]. Bowtie was run using default parameters with the modification that each read was allowed up to 3 mismatches. Furthermore, paired-end reads where one or both reads mapped to multiple locations were excluded from the analysis. The output from read mapping was stored in standard SAM format (http://bowtie-bio.sourceforge.net/manual.shtml#sam-bowtie-output) which indicates the contig, position and orientation for each mapped read and its mate. A perl pipeline was then constructed to convert this file into the required BAMBUS input files (http://sourceforge.net/apps/mediawiki/amos/index.php?title=Bambus_Manual). BAMBUS was then run using only the sequenced-based data and the output statistics collected (Table 1).

### Combining both the nonsequence and sequence based data

**Figure 3:**
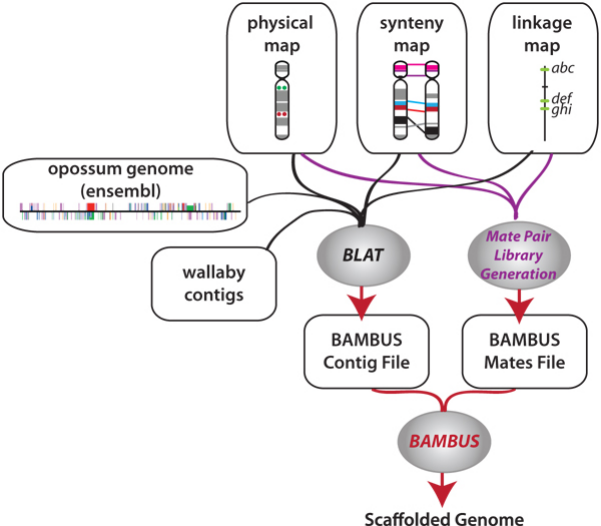
**Analysis pipeline.** This schematic is a representation of the steps in preparing the non-sequence based data for analysis by Bambus. The shaded ovals represent the programs and scripts, the rectangles represent the data sets while the arrows represent the flow of information through the pipeline.

Using the methods described above, the files from the sequence and non-sequence based data were combined and used together to further enhance the assembly. Given that the sequence based data is likely to be more precise than the non-sequence based information, a higher priority was assigned to it. Bambus will then use the sequence data to override the map data when there is a conflict between the two datasets. The statistics were again retrieved from the Bambus output statistics file and compared to that from just the map based or sequence based data alone. A diagram of this pipeline is shown in figure 3.

## Results

### Integrating the nonsequence-based data

From the 14,429 markers, 173,294 pseudo paired-end reads were generated across 6 libraries ranging from 5MB to 10MB intervals. The pseudo paired-end libraries increased the N50 from 36 KB in the original assembly, to 39 KB and included 2.2 % initial contigs in scaffolds. Based on the map-enhanced assembly alone, Bambus estimates the total scaffold span at 3.2GB, slightly larger than its predicted 2.7 GB size (A. Pask, personal communication).

### Integrating the sequence-based data

Using the 0.5× paired-end Illumina sequence data the genome was greatly improved, Bambus was able to order and orient 53% of the initial contigs into scaffolds and increase the N50 to 78KB. Interestingly, despite fewer reads, the 4 KB Illumina paired-end data was more successful in increasing the number of contigs in scaffolds compared to the 8 KB data (40% compared to 27%); however the 8KB data integration produced a larger N50 size (52 KB compared to 49 KB for the 4kb library alone) (Table 1).

### Combining both the non and sequence based data

Using both sequence and non-sequence based data, we were able to increase the number of initial contigs in scaffolds to 153,612, scaffolding 55% of the contigs from the original assembly. The inclusion of the mapping data to the Illumina paired-end libraries further increased the number of initial contigs being scaffolded by 5191. Thus, very few of the original 6024 initial contigs that the mapping data was able to include in a scaffold, were included using the Illumina paired-end libraries. Furthermore, the N50 for the assembly was greatly increased (by 35%) by the inclusion of the mapping data with the Illumina data (105 KB compared to 78 KB with Illumina data alone).

Since the N50 statistics for each of the analyses above were determined by using the total scaffold span for each analysis, they are not directly comparable. However, the total scaffold span for each analysis (with the exception of the use of the virtual map alone) is within the margin of error for our direct estimate of the genome size (2.7 GB +/- 10%; A. Pask personal communication). Furthermore, the comparatively small reduction in the estimated genome size concurrent with the inclusion of more paired end data, cannot alone account for the large increase in the N50 seen using this method.

## Discussion

Genome assemblies enhanced with non-sequence-based information (especially for low coverage genomes), provide a more workable resource for analysis and comparative genomics. Our bioinformatics pipeline provides a flexible, straightforward method of integrating non-sequence based data seamlessly into a modern genome project. The combination of, and ability to prioritize, sequence and non-sequence based data into an assembly gives this method robustness not found by simply mapping the assembled contigs to a virtual genome map. This prioritization allows the paired-end data to override the physical map when encountering small segmental inversions unique to a species or to even an individual. A small proportion (553 markers) of the virtual genome map was defined by fluorescence *in situ* hybridization (FISH) mapping which can identify the location of a gene on a specific chromosome within a few megabases. To address this precision limitation of FISH mapping, we constructed pseudo paired-end libraries starting with 5MB intervals to avoid any possibility of misinterpreting the order of the markers. Our findings showed that while the non-sequence data only marginally helped to increase the number of initial contigs scaffolded together, it is able to greatly improve the N50 size. The small increase in initial contigs scaffolded is not surprising given that the total number of paired-end reads generated from the map points was 173,294 compared to 20,133,999 paired-end reads from the Illumina data. In total, the virtual map contributed less than 1% of the data points used in the combined assembly but added 4% of the contigs to a scaffold. Therefore the mapping data, even if utilizing a limited number of datapoints, can provide a useful means for increasing the N50. This is likely due to the interval between paired reads (5-10 MB), which far exceeds the current capabilities of next generation sequencing and can provide a higher order structure to the genome assembly. In addition, the mapping data allows direct assignment of the initial contigs to the chromosomes, providing valuable information beyond that of sequence data alone and further enhancing the accuracy of the final assembly.

## Conclusions

The method we describe herein provides a simple pipeline for the inclusion of non-sequence based data into a genome. Integrating data from more than one source (sequence based and map based) advances the robustness and confidence of any genome assembly. Map data is able to anchor contigs to chromosomes further improving the genome assembly. While the integration of over 14,000 map points was only able to enhance the genome assembly by 2.2% in the tammar wallaby, its inclusion with Illumina paired-end data was able greatly increase the N50 of the genome (35% above that generated from the Illumina reads alone). Given the high cost and time commitment of constructing a physical map, we would not recommended the use of extensive FISH and linkage mapping to improve a genome assembly over generating a low coverage of paired-end data. However, as the number and diversity of genomes continue to increase in public databases, closely related genomes can be used at virtually no cost to generate extensive synteny maps. Such maps can be used in the method described here for increasing the size of scaffolds, allowing assemblies to span large stretches of repetitive DNA that paired-end libraries from the current next generation sequencing platforms are not able to cross. We suggest that, together with paired-end data, this novel method can greatly enhance the assembly of a low coverage genome project improving its utility for further analyses.

## Competing interests

The authors declare that they have no competing interests.

## Authors' contributions

TH designed the scripts and ran the analysis. JL generated the initial (tammar wallaby 1.2 genome) assembly and contigs for analysis. AP and RO performed the Illumina paired-end reads. CW constructed the virtual genome map data. TH, JL, RO and AP conceived the ideas and prepared the manuscript.
